# Relationship between Polyunsaturated Fatty Acid Metabolism and Atherosclerosis

**DOI:** 10.31083/j.rcm2504142

**Published:** 2024-04-09

**Authors:** Qiulei Liu, Zhaoxuan Liu, Ding Wu, Sheng Wang

**Affiliations:** ^1^Department of Vascular Surgery, Beijing Anzhen Hospital, Capital Medical University, 100029 Beijing, China; ^2^Department of Vascular Surgery, Central Hospital Affiliated to Shandong First Medical University, 250013 Jinan, Shandong, China

**Keywords:** atherosclerosis, polyunsaturated fatty acids metabolism, fatty acid desaturase gene cluster, fatty acid desaturase

## Abstract

Multiple factors cause atherosclerosis, meaning its pathogenesis is complex, and has not been fully elucidated. Polyunsaturated fatty acids are a member of the fatty acid family, which are critical nutrients for mammalian growth and development. The types of polyunsaturated fatty acids ingested, their serum levels, and fatty acid desaturase can influence the atherosclerotic disease progression. The fatty acid desaturase gene cluster can regulate fatty acid desaturase activity and further affect atherosclerosis. This study reviewed the research progress on the effects of polyunsaturated fatty acids on atherosclerosis regulated by fatty acid desaturase and the relationship between genetic variants of the fatty acid desaturase gene cluster and atherosclerotic cardiovascular disease.

## 1. Introduction

Atherosclerosis (AS) is a chronic inflammatory disease. Systemic or localized inflammation plays a central role in the onset and progression of AS, while inflammatory markers have been shown to predict cardiovascular disease (CVD) independently of traditional risk factors [1, 2, 3]. The downstream metabolites of polyunsaturated fatty acids (PUFAs), such as arachidonic acid (AA), prostaglandins (PG), thromboxanes (TXs), leukotriene (LTs), and other inflammatory factors, have an important impact on the development of AS. PUFAs and fatty acid desaturase (FADS), a key enzyme affecting its metabolism, play an equally important role in the pathogenesis of AS [4, 5].

As one of the essential dietary fatty acids in the human body, the content of PUFAs in the body reflects both the dietary intake and the fatty acid desaturase activity [5]. Previous studies have found that the type and amount of PUFAs being consumed are closely related to atherosclerotic cardiovascular disease (ASCVD). Prospective, observational studies support the role of omega-3 PUFAs in the primary prevention of ASCVD [6], although randomized controlled trials (RCTs) have often reached neutral conclusions [7, 8]. The potential impact of the intake of omega-6 PUFAs on ASCVD is also controversial, with previous studies suggesting that higher intakes of omega-6 PUFAs (predominantly linoleic acid) are associated with a lower risk of ASCVD [9, 10]. However, clinical studies have shown that excessive intake of ω-6 PUFAs (predominantly linoleic acid) leads to increased production of proinflammatory factors, which can lead to a higher risk of developing ASVCD [11, 12]. Thus, the roles of the omega-3 and omega-6 PUFAs in AS are complex and remain inconclusive. Studies on lipid metabolism disorders and inflammatory responses due to the regulation of gene expression have shown that there is as yet an undefined association between fatty acid desaturases, they are regulated by the FADS gene cluster, and AS, whereby fatty acid desaturase expression levels, as well as its activity, can differentially affect AS [13, 14, 15]. Based on existing studies, the effect of polyunsaturated fatty acid metabolism on atherosclerosis remains in the exploratory stage. This article aims to illustrate the relationship between polyunsaturated fatty acids, the regulation of fatty acid desaturases by the fatty acid desaturase gene cluster, and atherosclerosis.

## 2. Classification of Polyunsaturated Fatty Acids

Fatty acids are components of cell membrane phospholipids with specific functions, metabolism, and signaling roles. As a member of the fatty acid family, PUFAs are a crucial nutrient for mammalian growth and development, they are biologically active cellular components of membrane phospholipids, a substrate for signaling molecules, and a direct regulator of gene expression that can directly affect cellular function and the responsiveness of cells and tissues to signals [16, 17]. Moreover, PUFAs can regulate inflammatory processes by modulating signaling pathways [18, 19]. Fatty acids can be classified into short-chain, medium-chain, and long-chain fatty acids according to the number of carbon atoms they contain and into saturated fatty acids, monounsaturated fatty acids, and polyunsaturated fatty acids according to the number of carbon–carbon bonds they possess. PUFAs can be classified into two categories—ω-3 PUFA and ω-6 PUFA—according to the position of the double bond in their chemical structure and the principle of counting the position of the first double bond following the methyl carbon atom. The chemical structure of fatty acids is usually expressed as the number of carbon atoms, double bonds, and the position of the first double bond. For example, eicosapentaenoic acid (EPA) is expressed as 20:5 ω-3, meaning it contains 20 carbon atoms, five double bonds, and belongs to the ω-3 PUFA. In addition, fatty acids also have the Δ-coding system, which is different from the ω-coding system because the double bond position is counted from the carboxyl carbon atom.

## 3. Endogenous Metabolism of Polyunsaturated Fatty Acids

The human body cannot synthesize the amount of PUFAs needed for the body’s metabolism, meaning the ω-3 PUFA and ω-6 PUFA need to be supplemented through the diet, thus, they are referred to as essential fatty acids [20, 21]. The proportion of PUFAs in the diet is dominated by linoleic acid (LA) and α-linolenic acid (ALA), which are precursors of short-chain PUFAs that can be converted to biologically active long-chain PUFAs by Δ5 and Δ6 desaturase and elongase enzymes. The metabolism and metabolites of PUFAs will be summarized below according to the different types of PUFAs.

### 3.1 Endogenous Metabolism and Metabolites of Omega-3 Polyunsaturated Fatty Acids

**Fig. 1.**
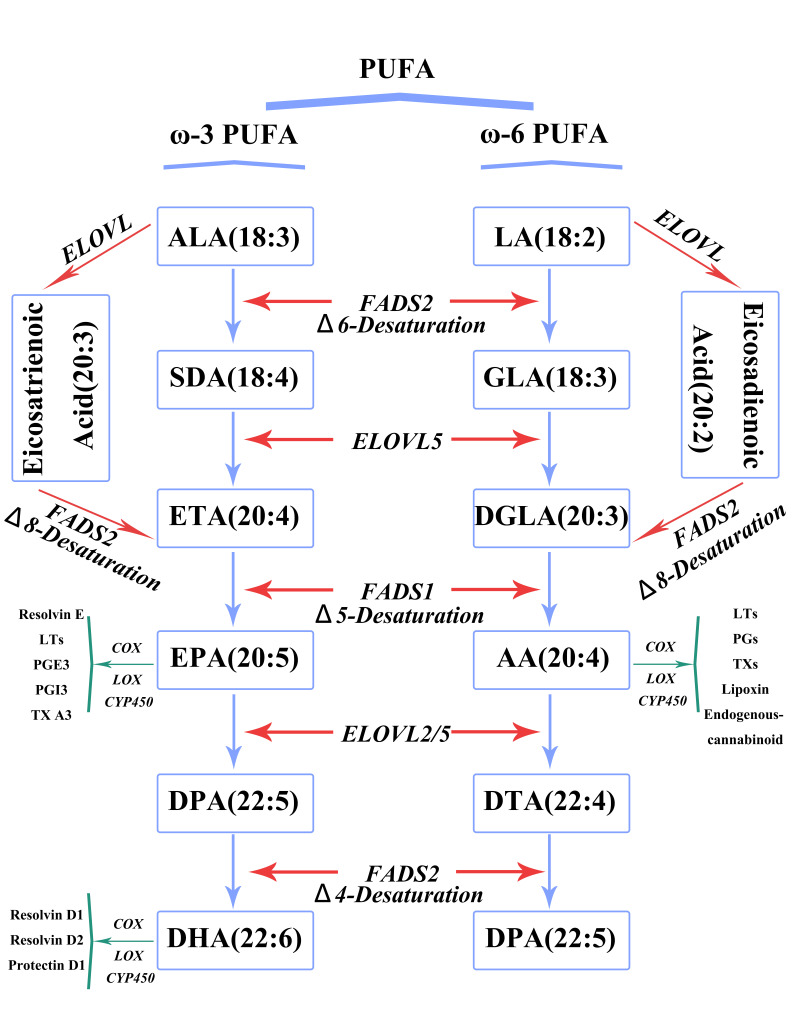
**Classification and metabolism of PUFAs**. ALA, α-linolenic acid; SDA, stearidonic acid; ETA, eicosatetraenoic acid; EPA, eicosapentaenoic acid; DPA, docosapentaenoic acid; DHA, docosahexaenoic acid; LA, linoleic acid; GLA, gamma-linoleic acid; DGLA, dihomogamma linoleic acid; AA, arachidonic acid; DTA, docosatetraenoic acid; DPA, docosapentaenoic acid; ELOVL, elongation of very long fatty acids; FADS, fatty acid desaturase; COX, cyclooxygenase; LOX, lipoxygenase; CYP450, cytochrome p450; LT, leukotriene; PGE3, prostaglandin E3; PGI3, prostacyclin I3; TX, thromboxane synthase; PUFAs, polyunsaturated fatty acids; FADS2, fatty acid desaturase 2.

ALA can be gradually converted to stearidonic acid (SDA), EPA, docosapentaenoic acid (DPA), and docosahexaenoic acid (DHA) *in vivo* by the action of the desaturase system and the elongation enzyme. The downstream metabolites of EPA and DHA are physiologically crucial for the organism. Specifically, EPA and DHA are catalyzed by cyclooxygenase (COX), lipoxygenase (LOX), and cytochrome P450 oxidase (CYP450) to produce a series of specialized pro-resolving mediators (SPMs). SPMs include separate families of molecules: resolvins, protectins, and maresins. These act as stimulatory cell agonists, arresting neutrophil infiltration and enhancing macrophage uptake of apoptotic cells [22]. Resolvins can inhibit neutrophil infiltration, inhibit platelet aggregation, and reduce the production of proinflammatory factors [23]. Protectins can promote the expression and activity of antiapoptotic proteins and inhibit the expression and activity of proapoptotic proteins [24]. In addition, EPA is catalyzed by COX to produce thromboxane A3, which inhibits platelet aggregation, prostacyclin I3, which promotes vasodilatation, as well as prostaglandin E3 and leukotriene LT5, which has anti-inflammatory properties. Thus, both EPA and DHA metabolites can exert anti-inflammatory effects (Fig. 1).

### 3.2 Endogenous Metabolism and Metabolites of Omega-6 Polyunsaturated Fatty Acids

LA can be converted to gamma-linolenic acid (GLA), double-high gamma-linolenic acid (dihomo-γ-linolenic acid, DGLA), arachidonic acid (AA), and DPA *in vivo*, under the action of the desaturase system and elongation enzyme. AA has essential biological functions and is catalyzed by COX to produce prostaglandin E2 and thromboxane A2, which promotes the inflammatory response, platelet aggregation, and vasoconstriction. AA can also produce proinflammatory factors from the leukotriene four-family, which, in the presence of LOX, play an essential role in the development and maintenance of the inflammatory response. In addition, AA is also catalyzed by LOX to produce lipoxin A4 (lipoxin) and lipoxin B4, which have a pro-resolving role in the abrogation of inflammatory responses (Fig. 1). However, in cardiovascular diseases, metabolites of AA exert deleterious effects that are proinflammatory, prothrombotic, and proplatelet aggregation to promote the development of atherosclerosis [25].

## 4. Effect of Polyunsaturated Fatty Acid Intake on Atherosclerotic Cardiovascular Disease

The role of PUFA intake in ASCVD has been controversial [6, 8, 26]. Although it has been demonstrated that ω-3 PUFAs can lower blood triglyceride levels and ω-6 PUFAs can lower blood total cholesterol levels, the results of the effects of ω-3 and ω-6 PUFAs on ASCVD in clinical practice have been inconsistent. Although clinical guidelines point to a positive effect from the use of icosapent ethyl and EPA in preventing ASCVD [27, 28, 29], there is a high degree of clinical heterogeneity in the design of previous studies and the final results. There is no solid evidence for using PUFAs to effectively treat or prevent ASCVD in patients with different backgrounds [30]. Therefore, the effects on ASCVD following the intake of PUFAs will be briefly summarized, as well as the reasons for the different results among the various studies.

### 4.1 Effect of Omega-3 Polyunsaturated Fatty Acids on Atherosclerotic Cardiovascular Disease

The controversy over the effect of ω-3 PUFAs on ASCVD lies in the fact that different clinical studies have yielded different results. REDUCE-IT, a multicenter RCT that included and followed more than 8000 patients with cardiovascular disease for almost five years, showed that compared to the placebo, patients who ingested EPA (4 g/d) underwent a significant reduction in the risk of cardiovascular death and nonfatal risk of myocardial infarction [31]. However, two similar RCTs (OMEMI (Omega-3 Fatty acids in Elderly with Myocardial Infarction), STRENGTH study (the Long-Term Outcomes Study to Assess Statin Residual Risk with Epanova in High Cardiovascular Risk Patients with Hypertriglyceridemia)) concluded that the intake of omega-3 PUFAs (EPA+DHA) did not reduce the risk of nonfatal myocardial infarction, stroke, or cardiovascular death [32, 33]. A systematic evaluation of the effects of ω-3 PUFAs on cardiovascular health showed that although the increased intake of EPA and DHA lowered plasma triglycerides and increased high-density lipoprotein (HDL) levels, they did not reduce the incidence of coronary artery disease (CAD), stroke, or other ASCVD events, nor the risk of death. In addition, increased intake of ALA reduces the risk of death from CAD, and increased intake of ALA has a preventive effect on ASCVD [34, 35, 36]. An RCT of the CVD population in the United States showed that increased omega-3 PUFAs (EPA+DHA) did not significantly reduce the risk of major cardiovascular events (myocardial infarction, stroke, and cardiovascular death) [8]. Circulating DHA, total omega-3, LA, and total omega-6 concentrations had no protective effect on the risk of cardiovascular disease in a Mendelian randomization study from the UK Biobank [37].

Although the above studies yielded different results, an analysis of these results from the different studies found that the composition of ω-3 PUFAs being consumed by the patients was different in each study, which may be one of the reasons for the controversy over the effects of ω-3 PUFAs on ASCVD. It has been suggested that EPA intake alone may be more effective in reducing the risk of cardiovascular disease than EPA+DHA [38], that serum EPA levels may need to reach a certain threshold to exert a preventive effect on ASCVD, and that a high dose (>1 g/d) significantly reduces the risk of cardiovascular events compared to low-dose EPA levels [39, 40]. In addition, when EPA was combined with DHA, higher DHA levels attenuated the preventive effect of EPA on ASCVD [30]. Therefore, the intake of EPA in ω-3 PUFAs and EPA blood levels in the study population may be important reasons for the observed variability in the effects of ω-3 PUFAs on ASCVD [41]. At present, the effect of omega-3 PUFAs on atherosclerosis remains a focus of clinical research. An ongoing RCT (NCT05365438) from Korea will assess the effects of combination therapy using atorvastatin and omega-3 PUFAs (EPA+DHA) compared with atorvastatin and ezetimibe combination therapy in diabetes mellitus type 2 (T2DM) patients with asymptomatic carotid atherosclerosis. The progression of carotid intima-media thickness and carotid artery plaques will be evaluated by three-dimensional (3D) carotid ultrasound. Another ongoing RCT (NCT05725486) from Croatia will investigate the influence of n-3 PUFAs enriched chicken on vascular and endothelial functions in a population of healthy young subjects and active athletes. Specifically, whether the intake of omega-3 PUFAs affects lipid profiles, oxidative stress, and inflammation. Further elucidating the mechanisms of vascular protection for omega-3 PUFAs may lead to new interventions for atherosclerosis in clinical practice.

### 4.2 Effect of Omega-6 Polyunsaturated Fatty Acids on Atherosclerotic Cardiovascular Disease

The effect of the intake of ω-6 PUFAs on ASCVD is equally controversial. A meta-analysis involving 44 prospective cohort studies showed that higher LA intake was associated with a reduced risk of death from CAD. It supported the potential long-term benefits of ω-6 PUFAs in reducing the risk of cardiovascular disease [9]. In addition, replacing a saturated fatty acid diet with an ω-6 PUFA (LA) may reduce the risk associated with CAD [42]. However, a systematic evaluation of the effects of ω-6 PUFAs on cardiovascular health suggests that increased intake of ω-6 PUFAs (LA+GLA) may reduce total cholesterol levels in the blood but does not significantly affect the risk of cardiovascular disease or mortality; therefore, the potential benefits in terms of reduced myocardial infarction remain to be demonstrated [43]. Another meta-analysis of randomized controlled trials showed that increasing the intake of omega-6 PUFAs (LA, GLA, DGLA, and AA) did not affect the incidence of myocardial infarction, stroke, CAD, and mortality [44]. An RCT studying the effects following the intake of different types of PUFAs from vegetable oils on cardiovascular disease in a population of hypercholesterolemic adults in China showed that after one year of measuring the intake of oleic acid (saturated fatty acid)-rich peanut oil, LA-rich corn oil, and ALA-rich blended oils, on fasting lipids, glucose, insulin concentration, and high sensitivity C-reactive protein levels of the different populations, the intake of different fatty acids did not affect cardiovascular risk factors [45].

Although some studies supported beneficial outcomes for cardiovascular disease following an increased intake of ω-6 PUFAs, several studies still produced conflicting results. The intake of ω-6 PUFAs may negatively impact ASCVD by causing an increase in downstream metabolites, such as proinflammatory 2-series prostaglandins and 4-series leukotriene. However, it is difficult to show a direct effect of ω-6 PUFAs on ASCVD when the impact of ω-6 PUFA metabolites on ASCVD is studied [42]. Therefore, more rigorous RCTs are needed to elucidate whether ω-6 PUFAs play a preventive or promotional role in ASCVD.

The competitive inhibition of enzymes between ω-3 and ω-6 PUFAs makes balancing the intake ratio between both ω-3 and ω-6 PUFAs complex. In addition, PUFA metabolism is also affected by genetic factors, which lead to alterations in the activity of fatty acid desaturase and the subsequent conversion of its products. Therefore, focusing only on the intake of a particular PUFA without considering the proportion of ω-3/ω-6 PUFAs in the diet and the influence of genetic factors on PUFA metabolism makes it difficult to explain the variability in the results from these studies.

## 5. Fatty Acid Desaturase and FADS Gene Cluster

### 5.1 Function and Classification of Fatty Acid Desaturases

The primary function of fatty acid desaturases is to dehydrogenate and introduce a double bond between the carbon atoms of the fatty acyl chain. In humans, membrane-bound fatty acid desaturases are known as “front-end” desaturases, and introduce a nascent double bond between an existing double bond, usually between the carboxyl group and the ninth carbon atom of the terminal methyl group, with front-end desaturation occurring at the Δ4, Δ5, Δ6, and Δ8 positions, while they are responsible for the endogenous biosynthesis of PUFAs. Therefore, fatty acid desaturases are categorized into four different types based on the location where desaturation occurs, namely, Δ4 fatty acid desaturases, Δ5 fatty acid desaturases, Δ6 fatty acid desaturases, and Δ8 fatty acid desaturases [46].

### 5.2 Structure of the Fatty Acid Desaturase Gene Cluster

**Fig. 2.**
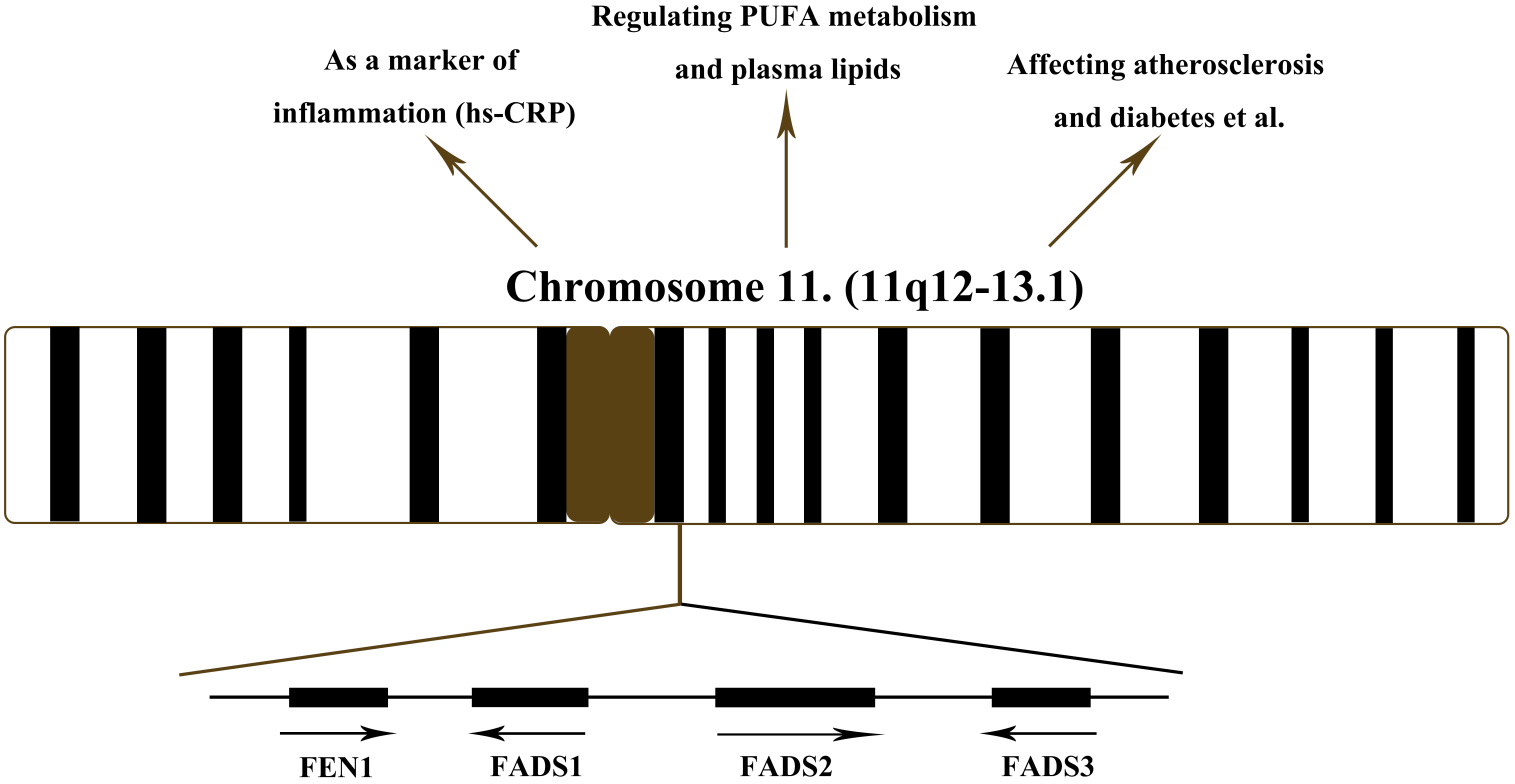
**Function of FADS cluster and location of FADS cluster in chromosome 11**. FEN1, flap endonuclease 1; FADS1, fatty acid desaturase 1; FADS2, fatty acid desaturase 2; FADS3, fatty acid desaturase 3; PUFA, polyunsaturated fatty acid; hs-CRP, high-sensitivity C-reactive protein.

The fatty acid desaturase (FADS) gene cluster that encodes fatty acid desaturase is located on human chromosome 11 (11q12-13.1) [47]. Data from the National Center of Biotechnology Information (NCBI) database demonstrates that *FADS1*, *FADS2*, and *FADS3* are composed of 13 exons and 11 introns, and the total lengths of *FADS1*, *FADS2*, and *FADS3* are 17.2, 39.1, and 18.7 kb, respectively (Fig. 2). *FADS1* encodes Δ5 fatty acid desaturase; *FADS2* encodes Δ4, Δ6, and Δ8 fatty acid desaturase; *FADS3* encodes Δ9 and Δ13 fatty acid desaturase. Since the gene clusters have the same location and similar structures, it is hypothesized that they have evolved based on gene duplication, meaning they have acquired substrate specificity [46, 48, 49].

### 5.3 Effect of Variants in the Fatty Acid Desaturase Gene Cluster on the Metabolic Activity of the Organism

Fatty acid desaturase is a critical enzyme in PUFA metabolism, and gene polymorphism in *FADS* affects the activity and function of fatty acid desaturase [50, 51], which in turn affects metabolic activities in the body, such as lipid concentrations, cardiovascular disease risk, pregnancy, cognitive function, Alzheimer’s disease, overweight, and type 2 diabetes mellitus [20, 52, 53].

The first exploratory study of variants in the FADS gene cluster found that single nucleotide polymorphisms (SNPs) in *FADS1* and *FADS2*affect the composition of serum phospholipid fatty acids in healthy adults, as evidenced by changes in the levels of LA, DGLA, AA, and DPA [54]. Other studies have obtained consistent results. Mid-FADS gene cluster variants in Caucasian populations are associated with higher levels of precursor PUFAs and lower levels of AA and EPA, less inflammation, and lower risks of cardiovascular disease [55, 56]. A recent cohort study on *FADS* SNPs and fetal growth and development in pregnant women in China’s Han population showed that pregnant women with the *FADS1*/*rs174448G*, *FADS3*/*rs174455T*, and *FADS3*/*rs174464A* allele should be supplemented with DHA-rich ω-3 PUFAs exogenously during pregnancy due to the blockage of the endogenous synthesis of DHA [57].

These findings suggest that FADS gene cluster variants have a direct impact on PUFA metabolism, which in turn affects the risk of inflammation and cardiovascular disease, and that FADS gene cluster variants in pregnant women have a direct effect on PUFA levels in breast milk, which in turn affects infant growth and development. Several studies on the association between variants in the FADS gene cluster and child development have shown that the *FADS* SNPs are strongly associated with the synthesis of DHA and AA *in vivo* and can influence intelligence quotient (IQ) as well as cognitive ability in infants and young children, as shown by the fact that genetic restriction of endogenous PUFA synthesis results in poorer cognitive development in infants fed formulas that do not provide DHA and AA. This developmental deficit can be eliminated when infants are fed AA. European legislation mandating the addition of AA and DHA to infant formula may address developmental deficiencies due to insufficient synthesis of endogenous PUFAs in infants or mutations in the FADS gene cluster [20]. A case–control study reported that the *FADS1*/*rs174556* genotype significantly increased the susceptibility to Alzheimer’s disease by regulating the efficiency of AA synthesis in ω-6 PUFAs [58]. However, this study had a small sample size and did not analyze AA derivatives in detail. Another case–control study reported an association between *FADS1*/*rs174556*, *FADS2*/*rs174617*, and obesity, by demonstrating that plasma levels of omega-6 PUFAs and AA were higher in overweight and obese patients; however, the difference in ω-3 PUFA levels was not significant. The study concluded that mutations in the *FADS1*/*FADS2* locus could cause metabolic disorders and increase the risk of cardiovascular disease [59]. Genome-wide association studies (GWAS) in European and Asian populations have shown that *FADS1*/*rs174546* is associated with reduced Δ5 fatty acid desaturase activity, obesity, and the risk of insulin resistance [60]. A study of the genetic etiology of type 2 diabetes mellitus showed that people with the *FADS1*/*rs174546G* allele had higher fasting insulin levels and higher HOMA-IR (an indicator to assess the level of insulin resistance) and that the increase in Homeostatic Model Assessment for Insulin Resistance (HOMA-IR) was more pronounced with elevated plasma DGLA and AA levels [61]. *FADS1*/*rs174547* effects on dyslipidemia have been reported in many races, and a study of the Chinese adult population showed that *FADS1*/*rs174547* was significantly associated with high triglyceride levels in men and negatively associated with low density lipoprotein (LDL) cholesterol levels in women, thereby suggesting that it may be a sex-specific SNP locus [62].

The FADS gene cluster affects a variety of diseases by regulating the metabolism of PUFAs. Genetic variants in the FADS gene cluster are associated with an increased risk of dyslipidemia, obesity, and insulin resistance. These outcomes are strongly associated with atherosclerotic disease and produce a state of hypersecretion of proinflammatory cytokines, which increases the body’s susceptibility to atherosclerotic disease.

### 5.4 Effect of Fatty Acid Desaturase Gene Cluster Variation on Atherosclerotic Cardiovascular Disease

Previous studies on the association between differences in dietary PUFAs and the metabolism of PUFAs in humans and ASCVD suggest that both ω-3 and ω-6 PUFAs and their downstream metabolites can affect ASCVD. However, there are relatively few studies on the effect of the FADS gene cluster on ASCVD.

Genetic studies have shown that variants in *FADS1*, encoding the Δ5 fatty acid desaturase, and *FADS2*, encoding the Δ6 fatty acid desaturase, are most directly genetically linked to plasma PUFA levels and that the FADS gene cluster is the most important locus for influencing PUFA metabolism [56, 63, 64, 65]. Therefore, the *FADS* SNP can alter the accumulation of PUFAs and directly affect ASCVD. Genetic studies have provided a theoretical foundation for exploring the impact of the FADS gene cluster on the pathogenesis of ASCVD. Several studies have found a strong association between FADS gene cluster variants and ASCVD (Table 1, Ref. [10, 51, 66, 67, 68, 69, 70, 71, 72, 73]). For example, a Mendelian randomization study of European and American populations showed that plasma ALA and LA levels were higher in the *FADS1*/*rs174547* sub-allele population and that this population was less likely to have CAD, stroke, and aortic stenosis, thereby suggesting that the *FADS1* SNP drives the association between plasma levels of PUFAs and ASCVD [66]. An RCT on the effect of the *FADS2* SNP on left ventricular remodeling after acute myocardial infarction (AMI) demonstrated that six months of high doses of ω-3 PUFAs after an AMI resulted in a significant attenuation of adverse left ventricular remodeling, noninfective myocardial fibrosis, and amelioration of the *FADS2 rs1535GG*-imposed hyperinflammatory response [51]. A case–control study in the Han Chinese population in northern China showed a strong association between the *FADS3* SNP and CAD. The recessive *G* allele of *FADS3 rs1000778* was associated with a higher risk of CAD, whereas the minor *AA* allele was associated with a lower risk of CAD; however, the plasma cholesterol and triglyceride levels remained similar between the two genotypes [67]. Aortic stenosis contributes to cardiovascular mortality and morbidity, and recent studies found that *FADS1* SNP is associated with the risk of aortic stenosis. A GWAS of 44703 participants in the Genetic Epidemiology Research on Adult Health and Aging (GERA) cohort, one of the largest collections of aortic stenosis in the world, demonstrated that the *FADS1*/*FADS2* locus variants (*rs174547*) are associated with aortic stenosis, while higher levels of AA and a higher ratio of AA/LA were associated with increased odds of calcification of the aortic valve leaflets [68]. A study of the relationship between localized PUFA in aortic valves and *FADS* genotypes by expression quantitative loci (eQTL) found that the minor *C* allele of *rs174547*, which corresponds to the protective genotype for aortic stenosis, was associated with higher *FADS2* mRNA levels in calcified valve tissues, whereas *FADS1* mRNA and other transcripts in proximity of the SNP were unaltered. In contrast, the Δ5 desaturase activity and the Δ6 desaturase activity were decreased. The authors concluded that the association between the *FADS1* genotype and lower risk for aortic stenosis may implicate DHA and DHA-derived specialized pro-resolving mediators that contribute to a protective effect [74]. In addition, the diet–gene interaction for ASCVD is crucial. A recent cross-sectional population-based cohort study demonstrated that differential associations between the *FADS1* locus variant and carotid–femoral pulse wave velocity (for assessing atherosclerosis) were observed depending on the intake of omega-3 PUFAs, with a high intake of omega-3 PUFAs attenuating the *FADS1* locus variant-dependent associations. This suggests that the high intake of omega-3 PUFAs (EPA/DHA) may compensate for an unfavorable *FADS1* locus genotype [75].

**Table 1.** **Studies on the association between FADS gene cluster variation and atherosclerotic cardiovascular disease**.

First author (year)	Study type	Intakes/evaluation indicators	SNP	Outcomes	Results
Baylin (2007) [69]	Case–control	ALA levels in plasma and adipose tissue	*FADS2* promoter deletion	MI	No significant effect
Kwak (2011) [70]	Case–control	Plasma PUFAs and total cholesterol levels	*FADS1 rs174537*	CAD	*FADS1 rs174537 T* allele decreased plasma total cholesterol, AA/LA ratio, and decreased the risk of CAD
Li (2013) [71]	Case–control	Δ6 fatty acid desaturase activity (AA/LA)	*FADS1 rs174537*	CAD	The *FADS1 rs174537T* allele population has lower Δ6 fatty acid desaturase activity and reduced CAD risk; however, the G allele has increased Δ6 fatty acid desaturase activity and increased CAD risk
Hellstrand (2014) [72]	Cohort	LA, ALA intakes	*FADS1 rs174546*	ASCVD	Negative association of dietary ALA: LA ratio or ALA intake with ASCVD in sub-allele T carriers
Liu (2015) [73]	Case–control	EPA, DHA intakes	*FADS1 rs174547*	CAD	Lower dietary intake of EPA or DHA individuals associated with a higher risk of CAD
Wu (2017) [67]	Case–control	Blood lipid level	*FADS3 rs1000778*	CAD	The secondary allele AA was associated with a lower risk of CAD, whereas the recessive allele *G* was associated with a higher risk of CAD
Marklund (2019) [10]	Meta	Plasma LA, AA levels	*FADS1 rs174547*	ASCVD	LA was negatively associated with ASCVD in carriers of the common allele in purebloods and not in carriers of the minor allele
Yuan (2019) [66]	Mendelian randomization	Plasma fatty acid levels	*FADS1 rs174547*	CAD, stroke, AS	The *FADS1 rs174547* sub-allele is negatively associated with ASCVD
Kwong (2019) [51]	RCT	ω-3 PUFAs intakes	*FADS2 rs1535*	Left ventricular remodeling after AMI	A high omega-3 PUFAs diet ameliorates the heightened inflammatory response associated with *FADS2 rs1535GG*, significantly attenuating adverse left ventricular remodeling and non-infarcted myocardial fibrosis
Chen (2020) [68]	GWAS	Plasma LA, AA levels	*FADS1*/*FADS2 rs174547*	Aortic valve stenosis and calcification	*FADS1*/*FADS2* locus variants are associated with aortic stenosis and calcification, and AA level is strongly associated with aortic stenosis

RCT, randomized controlled trial; GWAS, genome-wide association study; MI, myocardial infarction; CAD, coronary artery disease; ASCVD, atherosclerotic cardiovascular disease; AMI, acute myocardial infarction; AS, aortic valve stenosis; FADS, fatty acid desaturase; ALA, α-linolenic acid; PUFAs, polyunsaturated fatty acids; AA, arachidonic acid; LA, linoleic acid; EPA, eicosapentaenoic acid; DHA, docosahexaenoic acid; SNP, single nucleotide polymorphism.

The effect of FADS gene cluster variants on ASCVD is mainly due to regulating the metabolism of PUFAs in the body, by altering fatty acid desaturase activity. In addition, exogenous supplementation of PUFAs can reverse the adverse effects of certain FADS gene cluster variants, suggesting that certain *FADS* gene-deficient disorders can be treated by increasing the intake of PUFAs that can prevent or alter the course of cardiovascular diseases.

### 5.5 Exploratory Studies of the Effects of FADS1 on Atherosclerosis

Various clinical data indicate that the FADS gene cluster has an essential effect on PUFAs metabolism, ASCVD, and glucose metabolism. Since *FADS1* encodes Δ5 fatty acid desaturase and its metabolites, EPA and AA play essential roles in the inflammatory response and atherosclerosis. In a study investigating the effect of *FADS1* on atherosclerosis and its mechanism of action, Powell *et al*. [14] showed that under high-fat dietary conditions, *FADS1* knockout mice had weight loss, improved blood glucose, and reduced atherosclerotic plaques compared with wild-type mice. This study hypothesized that the low expression of *FADS1* is associated with a reduced inflammatory response in the arterial wall, which is mainly manifested as a reduced AA/LA ratio in plasma and adipose tissue, and plays a positive role in preventing AS. Shuichi *et al*. [76] found that oral administration of a Δ5 fatty acid desaturase inhibitor to apolipoprotein E (*ApoE*) KO mice on a high-fat dietary background resulted in a reduction in atherosclerotic plaques, a decrease in the levels of AA and DHA, and an increase in the levels of DGLA. This finding is in general agreement with Powell’s conclusions. Gromovsky *et al*. [15] used antisense oligonucleotides (ASOs) to specifically knockdown *FADS1* in the liver, adipose, and reticuloendothelial systems of low density lipoprotein receptor (*LDLR*) KO mice. However, they produced a different result, whereby the specific knockdown of the *FADS1* in *LDLR* KO mice aggravated atherosclerosis and the hepatic inflammatory response, which differed from the previous two studies. The authors highlight several possibilities for their differing conclusions. The study by Powell *et al*. [14] did not validate the hepatic levels of *FADS1* expression. Second, the Powell *et al*. [14] mouse model produced sub-alleles; therefore, the functionality of *FADS1* was not completely lost. In addition, the different genetic backgrounds of the two mice may be another reason for the inconsistent results. The study also noted that a diet of ω-3 PUFAs (ALA+SDA) reduced the area of the atherosclerotic plaque in the aortic root of the mice compared with a saturated fatty acid diet.

Studies have shown that mice of different genetic backgrounds present different outcomes after *FADS1* knockdown and that the percentage of PUFAs in the diet affects the survival and progression of atherosclerosis in mice. The downstream metabolites and signal transduction pathways regulated by *FADS1* have yet to be thoroughly studied. Whether *FADS1* can directly affect AS by affecting the production of inflammatory factors, such as PGs, LTs, and TXs is worthy of further exploration.

## 6. Fatty Acid Metabolism in Macrophages and Atherosclerosis

Monocytes/macrophages play a crucial role in the development and progression of ASCVD and the deterioration of advanced lesions. Intimal infiltration and modification of plasma-derived lipoproteins and their uptake, mainly by macrophages, with the ensuing formation of lipid-filled foam cells, can initiate atherosclerotic lesion formation and alter the efferocytotic removal of apoptotic cells and foam cells, which contributes to the progression of atherosclerotic lesions [77]. One of the properties of macrophages is the ability to dynamically regulate PUFAs metabolism during the acute phase of the inflammatory stimulus-response and inflammatory regression. This dynamic regulation of PUFAs metabolism may contribute to macrophage plasticity, in particular by controlling the balance between pro- and anti-inflammatory mediators [78]. Therefore, PUFAs metabolism in macrophages is closely related to atherosclerosis.

Liver X receptors (LXRs) are nuclear receptors that can participate in regulating cholesterol homeostasis and fatty acid metabolism and are essential in controlling inflammation and innate immune responses. A recent study investigating the treatment of human primary monocyte–macrophages with LXR agonists found that PUFAs synthesis was affected by the significant induction of Δ5 and Δ6 desaturases (*FADS1* and *FADS2*, respectively) after LXR agonist treatment alongside the induction of acyl-CoA synthase long-chain family member 3 (ACSL3) and the fatty acid elongase 5 (ELOVL5). In addition, LXR agonist treatment of ApoE-/- mice led to significant changes in the PUFAs profile in atherosclerotic arteries with increases in both the AA/LA and the DHA/EPA ratios, while also being associated with the decreased expression of proinflammatory genes, such as Cox2 and Il1β. Therefore, local production of PUFAs and derived lipid mediators in macrophages triggered by LXR within the atheromatous plaque could affect inflammation and the development of atherosclerosis [79]. Another study demonstrated that PUFAs can alter the micro RNA (miRNA) profiles of macrophages. When macrophages are enriched with either DHA or AA, they alter the expression of many miRNAs closely associated with inflammation, thus, suggesting that PUFAs are regulators of macrophage phenotypes and the inflammatory response [80]. Macrophages can sense internal and environmental changes and subsequently adapt their phenotype. This sequence is commonly named macrophage activation (classical or alternative) or polarization, and the most common are M1 and M2 polarization. *In vitro* stimulation of macrophages with interferon-gamma (IFNg) and lipopolysaccharide (LPS) leads to M1 polarization, while interleukin-4 (IL-4) or IL-13 treatment induces alternative activation of M2 polarization. M1 macrophages are characterized by a proinflammatory phenotype with intense bactericidal activities, while M2 macrophages are involved in the resolution of inflammation and in tissue remodeling and repair. The current view is that an unbalanced interplay between M1 and M2 macrophages could contribute to atherogenesis [81, 82]. Fatty acid synthesis in macrophages is activated during M1 polarization, and excess fatty acid and triglyceride synthesis promote foam cell formation with proatherogenic effects [83]. A study demonstrated that peroxisome proliferator-activated receptor gamma (PPARg), a nuclear receptor activated by fatty acid derivatives that control fatty acid oxidation, was required for M2 polarization [84]. A recent study found that *FADS1* knockdown in macrophages was associated with a tendency toward M1 and away from M2 polarization. Specifically, *FADS1* knockdown resulted in augmented LPS-driven proinflammatory gene expression yet was associated with diminished IL-4-driven alternative activation gene signatures. Therefore, *FADS1* reciprocally regulates M1 and M2 polarization programs in the macrophage [15]. The metabolism of fatty acids in macrophages involves M1 and M2 polarization processes, while *FADS1* affects the metabolism of PUFAs in macrophages, affecting the dynamic balance between proinflammatory and pro-resolving mediators. Future studies should focus on how macrophages regulate their metabolism of PUFAs, and more studies are needed to assess the impact of *FADS1* on atherosclerosis development under various metabolic conditions.

## 7. Conclusions

**Fig. 3.**
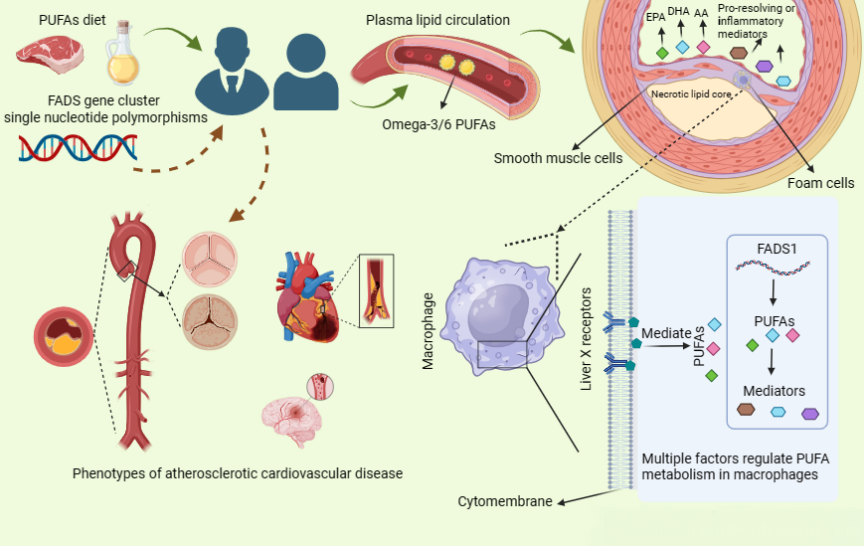
**The progression of atherosclerosis is associated with PUFAs intake, fatty acid desaturase, and FADS gene cluster variation**. Intake of polyunsaturated fatty acids has an important impact on atherosclerosis, and the effect of FADS gene cluster variants on ASCVD, mainly by regulating the metabolism of PUFAs in the body but more precisely by altering fatty acid desaturase activity. LXRs and *FADS1* expression regulate the metabolism of PUFAs in macrophages, while the metabolites of PUFAs in macrophages are also involved in the formation and progression of atherosclerosis. PUFAs, polyunsaturated fatty acids; FADS, fatty acid desaturase; ASCVD, atherosclerotic cardiovascular disease; LXRs, Liver X receptors; EPA, eicosapentaenoic acid; DHA, docosahexaenoic acid; AA, arachidonic acid.

Atherosclerosis results from a multifactorial combination of factors. The development and progression of atherosclerosis are associated with PUFAs, fatty acid desaturase, and FADS gene cluster variation (Fig. 3). Clinical trials to establish the necessary ω-3 to ω-6 ratio for optimum CVD health should consider ethnic background, genetic predisposition, biochemical markers, and dietary habits. The effects of dietary intake of ω-3 PUFAs and ω-6 PUFAs on atherosclerotic cardiovascular disease are controversial and need to be validated in more rigorous randomized controlled trials. Moreover, it is imperative to understand the differences between the impact of the formulation and the distinct effects of the ω-3/ω-6 PUFAs on lipid oxidation, inflammation, membrane structure/organization, cholesterol domain formation, and endothelial cell function. FADS gene cluster variants have an essential impact on atherosclerotic cardiovascular diseases and may be potential therapeutic targets to prevent and treat these diseases. *FADS* genotypes may be helpful for future stratification and targeting of dietary recommendations in individuals carrying the *FADS* minor allele. PUFAs and their metabolites are regulated by various factors, including FADS gene clusters and dietary background; thus, future in-depth studies of the association between PUFA and atherosclerosis will enrich the knowledge of the pathogenesis of atherosclerosis and provide new therapies for its treatment.
